# Ocular Vascular Changes in Mild Alzheimer’s Disease Patients: Foveal Avascular Zone, Choroidal Thickness, and ONH Hemoglobin Analysis

**DOI:** 10.3390/jpm10040231

**Published:** 2020-11-15

**Authors:** Elena Salobrar-Garcia, Carmen Méndez-Hernández, Rosa de Hoz, Ana I. Ramírez, Inés López-Cuenca, José A. Fernández-Albarral, Pilar Rojas, Surina Wang, Julián García-Feijoo, Pedro Gil, Juan J. Salazar, José M. Ramírez

**Affiliations:** 1 Instituto de Investigaciones Oftalmológicas Ramón Castroviejo, Universidad Complutense de Madrid (UCM), 28040 Madrid, Spain; elenasalobrar@med.ucm.es (E.S.-G.); rdehoz@med.ucm.es (R.d.H.); airamirez@med.ucm.es (A.I.R.); inelopez@ucm.es (I.L.-C.); joseaf08@ucm.es (J.A.F.-A.); pilar.rojas.lozano@gmail.com (P.R.); jgarciafeijoo@hotmail.com (J.G.-F.); 2 Facultad de Óptica y Optometría, Departamento de Inmunología, Oftalmología y ORL, UCM, 28037 Madrid, Spain; 3 OFTARED-ISCIII, 28040 Madrid, Spain; cdmendezh@gmail.com; 4 Hospital Clínico San Carlos, Institute of Health Care Research of the Hospital Clínico San Carlos (IdISSC), 28040 Madrid, Spain; su0507@outlook.com; 5 Facultad de Medicina, Departamento de Inmunología, Oftalmología y ORL, UCM, 28040 Madrid, Spain; 6 Hospital General Gregorio Marañón, Instituto Oftálmico de Madrid, 28007 Madrid, Spain; 7 Unidad de Memoria, Servicio de Geriatría, Hospital Clínico San Carlos, 28040 Madrid, Spain; pgil@salud.madrid.org

**Keywords:** Alzheimer’s, eye vascular network, choroid, retina, OCT, OCTA, Laguna ONhE, optic nerve head hemoglobin

## Abstract

In Alzheimer’s disease (AD), vascular changes could be caused by amyloid beta (Aβ) aggregates replacing the contractile smooth musculature of the arteriole walls. These changes happen in the brain vascular network, but also in the eye, and are related to decreased vascular density and low blood flow. In patients with Alzheimer’s disease, thinning of the choroid and the retina has been shown. The aim of this prospective study was to assess the retinal and choroidal vascular systems, analyzing the choroidal thickness with optical coherence tomography (OCT), the foveal avascular zone (FAZ) with OCT-angiography (OCTA), and the optic nerve head (ONH) hemoglobin with the Laguna ONhE program, to evaluate which of the two ocular vascular systems shows earlier changes in mild AD patients. These patients, compared to controls, showed a significantly thinner choroid at all the analyzed points, with the exception of the temporal macula (at 1000 and 1500 µm from the fovea). On the other hand, the FAZ and ONH hemoglobin did not show significant differences. In conclusion, a thinner choroid was the main ocular vascular change observed in mild AD patients, while the retinal vessels were not yet affected. Therefore, choroidal thickness could be used an early biomarker in AD.

## 1. Introduction

Cerebral amyloid angiopathy, present in 90% of patients with Alzheimer’s disease (AD), is one of the earliest pathological signs of the development of the disease [1,2]. In this vascular pathology, amyloid beta (Aβ) aggregates replace a significant amount of the contractile smooth musculature of the arteriole [3] and medium and small caliber artery walls [4,5,6,7]. The loss of smooth muscle cells disrupts the normal functioning of the neurovascular unit, with the regulation of local blood flow worsening due to a loss of vascular contractibility and a partial occlusion in the smaller distal arterioles by Aβ aggregates [8]. These changes have been associated with decreases in vascular density, vascular thickness, and blood flow in the brain [9]. 

The cerebral and retinal vascular networks show similarities [10,11]. In patients with AD, it has been speculated that retinal vascular changes share pathogenic mechanisms with those of brain vessels [4,11,12,13,14,15]. Therefore, these changes can be used for the study and monitoring of central nervous system (CNS) diseases. However, it should be taken into account that the ocular vascular system is derived from two systems that differ in their regulation and perfusion pressure, and could respond differently to a pathological process [16]. In retinal vascularization, the inner retina is supplied by blood vessels derived from the central retinal artery (CRA) [16], and its capillaries have a high 1:1 endothelial/pericyte ratio [17] that helps to regulate the blood supply. However, the outer retina is nourished by the choriocapillaris of the choroid [16]. In order to facilitate the passage of nutrients into the retina, the pericytes do not surround the capillary in this choroidal layer, appearing only towards the scleral side at an endothelial cells/pericytes ratio of 6:1 [18]. Given the contractile capacity of the pericytes, the low proportion of pericytes in the choriocapillaris might suggest that in the regulation of the choroidal blood flow, other regulatory mechanisms would intervene, such as the autonomic nervous system [18,19]. 

On the other hand, optic nerve perfusion depends not only on blood flow and oxygen saturation, but also on the Hb content of the optic nerve vessels. Changes in optic nerve head (ONH) reflectance can detect variations in Hb levels, indirectly allowing the assessment of ONH perfusion [20,21]. Laguna ONhE is a program that allows the measurement of ONH perfusion, detecting the pallor caused by a decrease in capillary density and, consequently, axonal loss [22]. Several studies have shown its usefulness in both ocular [22,23,24,25,26] and cerebral [27,28] neurodegenerative diseases.

In patients with AD, thinning of the choroid and the retina has been shown mainly by using optical coherence tomography (OCT) [29,30]. Taking into account the particular characteristics of each vascular system (the ciliary system and the CRA system), in this study of mild AD patients, we analyzed both systems using complementary tools—(i) the choroidal thickness using OCT, (ii) the foveal avascular zone using OCT angiography (OCTA), and (iii) the hemoglobin (Hb) concentration of the optic nerve using ONH photographs with the Laguna ONhE program—in order to elucidate which of the two vascular systems responsible for ocular irrigation begins to deteriorate earlier, and to provide earlier information for the diagnosis of the disease. 

## 2. Materials and Methods

### 2.1. Subjects 

In this prospective study, patients from among 3411 subjects in the Database of the Memory Unit of the Hospital Clínico San Carlos in Madrid (Spain) were included. Patients included in this work had a Mini Mental State Examination (MMSE) above 21 and a diagnosis of mild cognitive impairment according to the Clinical Dementia Rating scale (CDR). The ophthalmic medical records of these patients were reviewed, excluding those who were previously diagnosed with an ophthalmological pathology (retinal diseases, media opacity, and glaucoma or suspected glaucoma). Healthy individuals from the Geriatric Unit of the Hospital Clínico San Carlos (Madrid), or relatives of AD patients (husband or wife) with an MMSE >25 and no memory impairment, were selected as control subjects. Written informed consent was obtained from both groups. All patients were in possession of all relevant facts to give written informed consent. The protocol was approved by the local ethics committee (CEIC Hospital Clínico San Carlos 11/372-E), and followed the tenets of the Declaration of Helsinki. 

The clinical evaluation of patients with AD was described in our previous work [31], and the procedure is described in Table 1.

### 2.2. Optical Coherence Tomography (OCT) for Choroidal Thickness Analysis 

After pharmacological mydriasis, the choroidal thickness and the retinal foveal avascular zone (FAZ) were measured by the OCT Spectralis (Heidelberg Engineering, Heidelberg, Germany), which has good reproducibility and repeatability [32]. The retinal nerve fiber layer (RNFL) thickness was scanned three consecutive times per patient in each area studied. Areas selected for RNFL thickness measurement were scanned three times by the same optometrist (ESG), and under similar conditions, in each patient. Good-quality scans were those with a signal-to-noise ratio >30, and 95% accepted B-Scans. Statistical analysis was carried out with the means of the global thickness values.

**Figure 1:**
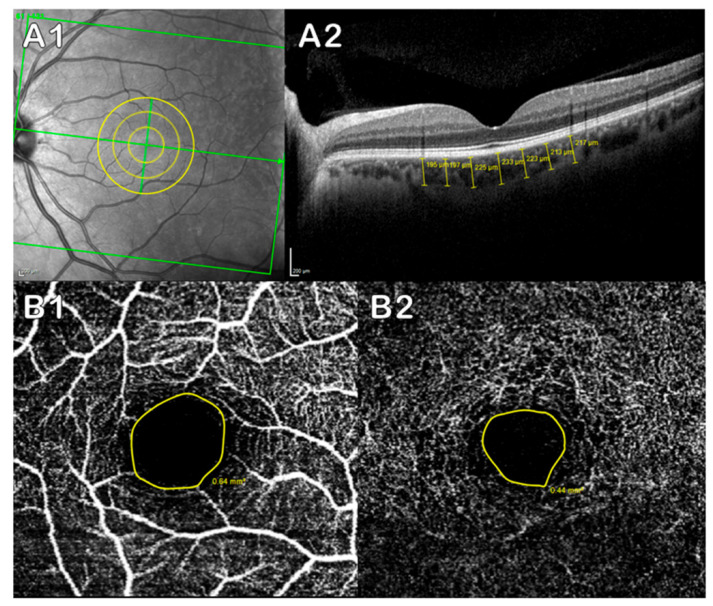
Measurement of the choroidal thickness and Foveal Avascular Zone (FAZ). (**A**) Spectralis OCT analysis of choroidal thickness: (**A1**) retinal zone analyzed; (**A2**) choroidal thickness quantification (μm). (**B**) OCTA analysis of the FAZ; yellow circle delimiting the avascular area of each plexus: (**B1**) superficial capillary plexus; (**B2**) deep capillary plexus.

The choroidal thickness was evaluated manually with the measurement function provided by the software itself. The same examiner delimited the choroidal thickness perpendicular to the retina from the outer hyper-reflective line of the retinal pigment epithelium (RPE) to the choroidal–scleral interface (CSI). Subfoveal measurements were performed in the superior, nasal, inferior, and temporal sectors. In each of the sectors, three measurements were made, each one separated by 500 μm; the measurements were made at 500, 1000 and 1500 μm from the center of the fovea. We considered the macula center as the thinnest point of the fovea (Figure 1A). The FAZ area was measured for both the superficial and deep capillary plexus of the macula using the Spectralis OCT angiography (OCTA) module. For this, the macular OCTA was taken into account, and a manual delimitation of the avascular area of each plexus was performed with the area tool provided by the software (Figure 1B).

### 2.3. Optic Nerve Head (ONH) Colorimetric Assessment

Two photographs of each eye fundus were obtained using the non-mydriatic retinal camera Canon CR-DGi (Canon Inc., Tokyo, Japan). Optic nerve head (ONH) colorimetric evaluation determined the ONH Hb amount using the Laguna ONhE program, as described in previous studies [20,22,25]. The percentages of the amount of ONH Hb were quantified blindly and independently of the examiner, since the value is automatically provided by the Laguna ONhE program. This software uses algorithms to divide the optic disc into 24 zones, or sectors, defining three concentric rings sectioned by eight radii, and automatically identifies the central optic nerve head vessels. Thus, two different zones were differentiated; one corresponding to the central ONH vessels (artery and vein) and one of the ONH tissue itself. All images that did not pass the quality control of the Laguna ONhE program, or images of poor quality due to media opacity or inadequate focus, were excluded.

The Hb levels present in the different tissue zones are expressed as a percentage relative to the maximum value [22,25]. 

Tissue Hb levels diminish from the disc periphery towards the center and, accordingly, colors change proportionally such that Hb levels can be topographically determined. 

The colorimetric representation of the changes of ONH Hb between patients with mild AD and controls was done with excel software and the color scale function. The values taken into account for this scale were 0, in white, when there was no difference, −10, with a blue tone, for when the retina was thinner, and +10, with a red tone, when the retina was thicker in patients with mild AD compared to controls. The program automatically provides the tone according to the variation in thickness.

### 2.4. Statistical Analysis

Statistical analysis was performed using SPSS 25.0 (SPSS Inc., Inc., Chicago, IL, USA). The data were reported as medians (interquartile range). The differences between different study groups (mild AD and control eyes) were analyzed using the Mann–Whitney test. A *p*-value of ≤ 0.05 was considered statistically significant.

### 2.5. Data Availability

The datasets analysed during the current study are available in the Figshare repository, https://dx.doi.org/10.6084/m9.figshare.12328340.

## 3. Results

In the mean Mini Mental State Examination (MMSE) values, there were significant differences between the study groups (control vs. mild AD) (*p* < 0.05) (Table 2, Table 3 and Table 4). There were no differences in sex, age, or education level between groups (*p* > 0.05).

### 3.1. Choroidal Thickness

For the study of choroidal thickness, 17 mild AD patients and 15 controls were included. Data are shown in Table 2. In the choroid of mild AD patients, there was a significant decrease in thickness in the subfoveal analysis; in all analyzed nasal, superior and inferior points (500, 1000 and 1500 µm away from the fovea); and in the inferior analysis at 500 µm away from the fovea (*p* < 0.05; in all instances).

### 3.2. Foveal Avascular Zone (FAZ)

For FAZ analysis, 14 mild AD patients and 18 control subjects were included. In the superficial FAV, there was a non-statistical difference (*p* > 0.05) between mild AD patients (0.71 (0.51–0.80) mm^2^) and the control group (0.54 (0.43–0.69) mm^2^). Additionally, in the deep FAZ, there was no difference between the mild AD group (0.29 (0.22–0.46) mm^2^) and controls (0.28 (0.19–0.36) mm^2^) (Table 3). 

### 3.3. ONH Colorimetric Assessment

**Figure 2:**
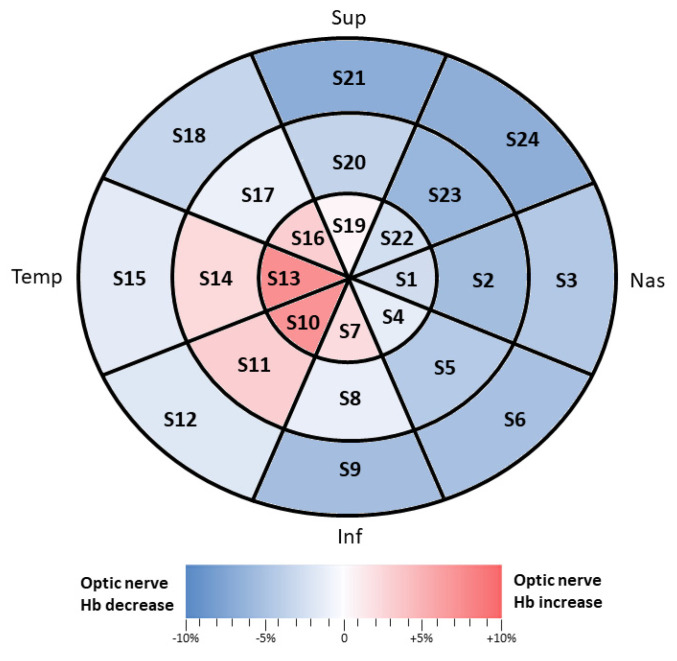
Colorimetric percentage differences of hemoglobin in the optic nerve head, analyzed using photographs of the optic nerve with the Laguna ONhE program, between the mild AD and control groups. Blue tones: decreased hemoglobin in patients with AD compared to the control group. Red tones: increased hemoglobin in patients with AD compared to the control group. Abbreviations: Sup (superior), Inf (inferior), Temp (temporal), Nas (nasal), S (sector).

Sixty-six eyes were included for ONH Hb analysis in this study, from 17 patients with mild AD and 49 controls. All data are shown in Table 4 and Figure 2. In none of the parameters analyzed were there any significant differences (*p* > 0.05) in the Hb values in the ONH.

## 4. Discussion

The choroid is one of the most vascularized tissues of the body, and its main function is to provide nutrients and oxygen to the outer retina [33]. There is increasing evidence suggesting that the ocular vascular network, like that of the brain, is also altered in AD, leading to choroidal abnormalities in this pathology [34], although other researchers have not found differences between controls and patients with AD [35,36].

In our study, compared to controls, the choroid was thinner in patients with mild AD (MMSE 26.0 (21.5–29.0)). This thickness decrease was found in the subfoveal point, in all the nasal, superior and inferior analyzed points (from 500 to 1500 µm away from the fovea), and in the temporal point 500 µm from the fovea. Our results are consistent with previous studies [34,37,38,39], where reductions in choroidal thicknesses were also found in AD patients with a mean MMSE lower than in our patients. Furthermore, in these studies, patients with lower MMSE scores showed a thinner choroid at more examined points, sometimes even affecting the entire analyzed choroid [34,37,38,39], while, on the other hand, other published works performed in MCI or early AD patients have shown no alteration in choroid thickness [35,36]. In the only follow-up study conducted, the choroids were analyzed again after 12 months, finding that thinning was more prominent in patients with AD than in controls. Similar results of a decrease in choroidal thickness have also been observed in histopathological studies in a rat model with AD, and in postmortem human samples from donors with AD [40]. 

The thinner choroid in AD could be associated with hypoperfusion and/or atrophic changes in this ocular vascular layer. These pathological changes in the vascular network could be caused by local Aβ deposits, similar to those found in the cerebral vascular system in AD patients [39]. Previous studies have demonstrated accumulations of Aβ in choroidal vessels in a normal aging mouse model and in a transgenic mouse model of AD [41,42]. As in the brain, the accumulation of Aβ in the choroid could induce an inflammatory response and activation of complements, which progressively leads to neurodegeneration and vasoregression of choroidal vascularization through the same pathological cascade that has already been described in brains with AD [40,43,44,45]. Thus, the choroidal changes observed in our study in patients with mild AD could reflect the importance of vascular factors in the pathogenesis of AD [34], and could contribute to the study of the severity and progression of the disease.

In the retina of patients with AD, it has been shown that there is vascular narrowing and a reduction in blood flow [13,46,47,48,49], suggesting a correlation between the flow density of the macula and vascular cerebral lesions [49]. In our study, when analyzing both the superficial and deep FAZ, no significant differences were found between patients with mild AD and controls. Currently, there are few studies where the surface of the FAZ has been analyzed in patients with AD [10,36,50,51,52]. Two of these studies were performed with the Optovue OCTA [10,50], and they showed a significant area increase in the FAZ. In one of them [10], the stage of the evolution of AD was much greater than in our study, with an MMSE score of 16.92 ± 7.39 (our study had an MMSE score of 26.5 (24.0–30.0)), and in the other study, this value was not indicated [50]. According to our results with the Spectralis OCTA, both using the RTVue XR OCTA and Cirrus 5000 Angioplex, no differences in the FAZ in either the superficial or the deep layer were found in early AD patients [36,49]. It must be considered in the interpretation of these results that the enface OCTA allows the precise and reliable identification of arteries and veins, while for small branches and crossings, identification by OCTA can be less precise and reliable [53]. In an OCTA study of the vascular plexus around the ONH in early AD patients, a reduced vessel density in the radial peripapillary capillaries layer was found, showing that vascular alterations are not limited to the macula [49].

In our work, it should be noted that the absence of significant changes in the FAZ of our patients with mild AD was accompanied by hemoglobin values in ONH, without significant differences with respect to control patients in any of the parameters analyzed. However, as observed in patients with greater cognitive impairment, when the disease progresses, vascular changes related to AD may become visible in the retinal vascularization [10,50]. 

In most tissues, blood flow tends to remain constant despite moderate variations in perfusion pressure, thanks to self-regulation mechanisms [54]. Retinal circulation lacks autonomic innervation, shows efficient self-regulation, and is mainly influenced by local factors produced by a combination of metabolic and myogenic mechanisms [55,56]. However, choroidal circulation is mostly not controlled by any self-regulation mechanism [55,57,58,59,60,61,62,63], and instead it is mainly controlled by autonomous and sensitive innervation [56]. In the choroid, there is a large number of nerve fibers [55]. These fibers are more numerous in the central choroid, especially in the submacular region, than in the periphery [64]. Likewise, sympathetic choroidal ganglion cells that are positive for neuropeptide Y (NPY)) and tyrosine hydroxylase (TH) were most frequently observed in the central region of the choroid [65]. The reason for a higher density of nerve fibers and choroidal ganglion cells in the submacular choroid could be because these neurons contribute to the regulation of arteriolar blood flow in this area [55]. It has been demonstrated in experimental works that sympathetic innervation is key to the regulation of choroidal vascularization, and that chronic loss of this sympathetic regulation can contribute to abnormal vascular regulation in diseases such as age-related macular degeneration (AMD) [66]. Thus, in AD, a pathology that many studies have related to AMD [67], it could be possible that choroidal vascular alterations could occur before retinal ones. 

Microcirculation in the brain has been extensively studied in AD. Although small vessel disease is implicated in the development and progression of AD [68], the brain microcirculation is very difficult to assess in vivo. The small blood vessels of the retina and brain share an embryological origin, anatomical features, and physiological properties (for example, non-anastomotic end arteries, and barriers) [11,69]. The vessels of the retina, which are 100–300 mm in size, allow non-invasive in vivo visualization of human microcirculation, providing a unique and easily accessible “window” for studying cerebral microvascular pathology [46].

Optic nerve perfusion depends on three factors, namely, oxygen saturation, blood flow, and Hb content. It has been suggested that changes in ONH reflectance can detect variations in Hb levels, which may be useful for indirectly measuring ONH perfusion [20,21]. In this study, the Laguna ONhE software was used to analyze the color changes in the ONH using Hb as a reference pigment, compensating for different variables such as illumination or lens absorption and diffusion. The purpose of this program is not simply to measure the perfusion of the ONH, but also to detect the paleness caused by a decrease in capillary density and, consequently, axonal loss [22]. It has been shown that the Laguna ONhE software has great precision and reproducibility in early diagnosis in patients with incipient glaucoma compared to the classic functional and structural tests used for this purpose [22,23,24,25,26]. Other studies have reported its usefulness in neuro-ophthalmic pathology, such as multiple sclerosis [27] or Parkinson’s disease [28], in which axonal loss also occurs. 

In our study, no significant differences were found in Hb measurements in the ONH between patients with mild AD and controls. In patients with mild AD, there were areas with less optic nerve Hb, especially in the nasal area (in the outer ring in S24 and S3, the intermediate ring in S23 and S2, and the inner ring in S22 and S1). In addition, areas with an increase in optic nerve Hb were found in the temporal region (in the intermediate and inner rings); the largest increase was found in S10 and S13. 

The only study published to date in patients with AD using this software showed a significant decrease in optic nerve Hb in the ONH in the same sectors in which we found a non-significant decrease. In addition, in the areas where they observed a non-significant decrease, our study showed a non-significant increase in optic nerve Hb [70]. This study [70] was performed in patients with an MMSE score of 16.56. However, our study was conducted in patients at a milder stage of the pathology, with an MMSE of 23 (21–26.5). The data from our mild AD patients could correspond to more initial changes than those observed by Bambo et al. [70], because our patients presented with better values of MMSE. In our patients with mild AD, it seems that papillomacular bundle perfusion was not yet affected. Bambo et al. [70] observed that the low hemoglobin levels in the neuroretinal ring of patients with AD did not cause a direct structural consequence, so the peripapillary thickness was unchanged. Thus, they postulated that in AD, ischemia of the optic disc must occur in a chronic and stable manner, and for this reason it may be better tolerated by the optic nerve [70]. 

One of the strengths of this study was the analysis of the ocular vascular network using three different but complementary tests. It must be taken into account that a weakness of this study is that vascular density was not analyzed. It is necessary to perform the analysis of this parameter in initial AD patients carefully selected by very strict criteria. Another limitation of our study is that it is not a longitudinal study. It must be taken into account that studies in AD patients are very difficult to carry out due to the particular characteristics of these patients and their elderly age. However, longitudinal studies that analyze the vascularization of these patients will be necessary in order to better understand the progression of vascular changes in AD patients. 

## 5. Conclusions

In conclusion, in our mild AD patients, the main change in the ocular vascular network was a thinner choroid, as measured by OCT. However, the retinal vessels were not yet affected, as demonstrated in the FAZ by OCTA and in the analysis of ONH hemoglobin by the Laguna ONhE software. Therefore, choroidal thickness could be an early biomarker in AD. 

## Figures and Tables

**Table 1:** Clinical evaluation of AD patients and ophthalmological inclusion criteria.

Clinical Evaluation of AD Patients		
Geriatrician Evaluation	Ophthalmological Evaluation	
	Tests	Inclusion criteria
Review of medical reports	Refraction	< ± 5 spherocylindrical
Caregiver interview	Biomicroscopy	AREDS < 2
Physical and neurological examinations	Visual acuity	>0.5 dec
Psychometric test MMSE	Intraocular pressure	<21 mmHg
Neuroimaging techniques	Color fundus photography	Free of ocular disease
Routine laboratory testing for dementia	OCT/OCTA	Good quality scans with no artefacts

(AD: Alzheimer’s Disease; AREDS: Age-Related Eye Disease group; MMSE: Mini Mental State Examination; dec: decimal scale; OCT: optical coherence tomography; OCTA: OCT angiography).

**Table 2:** Median choroidal thickness and *p*-value in mild AD patients and the control group.

	Control	Mild AD	*p*-Value
	(*n* = 15)	(*n* = 17)	
MMSE	30.0 (28.75–30.0)	26.0 (21.5–29.0)	**0.026** *
Subfovea	257.0 (216.0–275.0)	201.5 (175.0–233.7)	**0.005** *
Temp 500 µm	253.0 (218.0–281.0)	197.5 (173.7–249.5)	**0.025** *
Temp 1000 µm	232.0 (207.0–278.0)	213.5 (182.2–240.2)	0.150
Temp 1500 µm	237.0 (189.0–272.0)	218.5 (186.2–249.2)	0.434
Nas 500 µm	232.0 (204.0–272.0)	195.0 (159.5–233.7)	**0.013** *
Nas 1000 µm	221.0 (198.0–261.0)	200.0 (169.2–216.2)	**0.040** *
Nas 1500 µm	213.0 (182.0–252.0)	186.0 (168.5–202.5)	**0.025** *
Sup 500 µm	248.0 (207.0–261.0)	208.5 (166.2–236.5)	**0.015** *
Sup 1000 µm	244.0 (202.0–273.0)	208.5 (165.5–241.0)	**0.032** *
Sup 1500 µm	237.5 (209.0–282.0)	219.0 (166.5–230.0)	**0.020** *
Inf 500 µm	241.5 (207.7–276.2)	199.5 (164.2–234.0)	**0.013** *
Inf 1000 µm	245.0 (214.2–288.0)	207.0 (169.7–236.2)	**0.014** *
Inf 1500 µm	251.5 (214.7–269.7)	213.5 (166.0–240.0)	**0.029** *

Median (interquartile range); * *p* < 0.05 (in bold), Mann–Whitney U test. (AD: Alzheimer’s disease; MMSE: Mini Mental State Examination; vs: versus; Temp: temporal; Nas: nasal; Sup: superior; Inf: inferior).

**Table 3:** Median Foveal Avascular Zone and *p*-value in mild AD patients and the control group.

	Control	Mild AD	*p*-Value
	(*n* = 18)	(*n* = 14)	
**MMSE**	29.0 (28.5–30.0)	26.5 (24.0–30.0)	**0.030** *
**Superficial FAZ (mm^2^)**	0.54 (0.43–0.69)	0.71 (0.51–0.80)	0.288
**Deep FAZ (mm^2^)**	0.28 (0.19–0.36)	0.29 (0.22–0.46)	0.357

Median (interquartile range); *p < 0.05 (in bold), Mann-Whitney U test (AD: Alzheimer’s Disease; FAZ: foveal avascular zone).

**Table 4:** Percentage of hemoglobin in the optic nerve head (ONH) analyzed using photographs of the optic nerve with the Laguna ONhE program in the mild AD and control groups.

		Control	Mild AD	Mild AD vs. Control	
		(*n* = 49)	(*n* = 17)	% Mean Variation	*p*-Value
**MMSE**		30 (30–30)	23 (21–26.5)		<0.001 **
**Total Hb**		66.1 (62.55–70.65)	64 (55.65–73.95)	6.65	0.608
**Sectors**	S1	72 (67.15–77)	72.2 (56.8–79.85)	1.46	0.792
	S2	71 (66.65–75.55)	67.1 (56.6–76)	1.59	0.259
	S3	65.5 (60.95–71.5)	60.7 (56.9–72.15)	3.30	0.352
	S4	70.9 (65.95–76.1)	71.5 (58.95–79.75)	2.70	0.849
	S5	70.8 (65.4–74.4)	69.3 (56.35–75.6)	0.79	0.495
	S6	67.1 (58.75–70.1)	63.9 (55.65–69.15)	2.61	0.253
	S7	69 (63.75–73.55)	68 (60.4–78.75)	−3.11	0.901
	S8	70 (64.2–73.7)	66.9 (58.7–77.8)	−1.36	0.764
	S9	67 (58.8–70.9)	62.6 (51.75–71.9)	0.69	0.235
	S10	66.1 (57.05–72.4)	69.2 (58.15–81.5)	3.21	0.229
	S11	66.4 (61.4–70.25)	67.3 (57.4–77)	−4.31	0.553
	S12	63.6 (57.9–69.1)	63.3 (53.7–72.15)	−1.84	0.775
	S13	65 (56.45–71.1)	69.3 (56.45–81)	1.08	0.226
	S14	66.3 (60.1–70.4)	69.2 (55.4–75.05)	−4.45	0.468
	S15	62.5 (58.05–68.2)	60.2 (53.8–71.6)	−1.47	0.843
	S16	66.5 (60.8–72.85)	69.1 (57.5–79.7)	0.86	0.66
	S17	68.3 (64.15–73.85)	66.9 (58.5–79.1)	−1.86	0.965
	S18	64.8 (59.9–68.2)	65.5 (56.15–72.55)	0.56	0.665
	S19	70.1 (65.3–76.35)	69.9 (61.4–82.1)	2.01	0.883
	S20	71.1 (66.5–75.95)	71.8 (60.1–77.8)	−0.33	0.538
	S21	64.8 (59.05–71.7)	61.9 (52.8–68.8)	2.14	0.168
	S22	71.4 (65.35–78.4)	70 (57.75–83.75)	4.12	0.764
	S23	70.7 (67.1–76.15)	68.3 (57.65–74.5)	1.52	0.235
	S24	65.7 (58.45–72.25)	62.4 (53.3–69.05)	3.66	0.177

Median (interquartile range); * *p* < 0.05 (in bold), Mann—Whitney U test (AD: Alzheimer’s disease; vs.: versus; S: sector).

## References

[1] de la Torre J.C. (2002). Alzheimer disease as a vascular disorder: Nosological evidence. Stroke.

[2] Bell R.D., Zlokovic B.V. (2009). Neurovascular mechanisms and blood–brain barrier disorder in Alzheimer’s disease. Acta Neuropathol..

[3] Attems J., Lauda F., Jellinger K.A. (2008). Unexpectedly low prevalence of intracerebral hemorrhages in sporadic cerebral amyloid angiopathy: An autopsy study. J. Neurol..

[4] Masuzzo A., Dinet V., Cavanagh C., Mascarelli F., Krantic S. (2016). Amyloidosis in retinal neurodegenerative diseases. Front. Neurol..

[5] Yamada M. (2015). Cerebral amyloid angiopathy: Emerging concepts. J. Stroke.

[6] Vinters H.V. (2015). Emerging Concepts in Alzheimer’s Disease. Annu. Rev. Pathol. Mech. Dis..

[7] Lai A.Y., Dorr A., Thomason L.A.M., Koletar M.M., Sled J.G., Stefanovic B., McLaurin J.A. (2015). Venular degeneration leads to vascular dysfunction in a transgenic model of Alzheimer’s disease. Brain.

[8] Thal D.R., Griffin W.S.T., de Vos R.A.I., Ghebremedhin E. (2008). Cerebral amyloid angiopathy and its relationship to Alzheimer’s disease. Acta Neuropathol..

[9] Zlokovic B.V. (2011). Neurovascular pathways to neurodegeneration in Alzheimer’s disease and other disorders. Nat. Rev..

[10] Bulut M., Kurtulus F., Gozkaya O., Erol M.K., Cengiz A., Akidan M., Yaman A. (2018). Evaluation of optical coherence tomography angiographic findings in Alzheimer’s type dementia. Br. J. Ophthalmol..

[11] Patton N., Aslam T., MacGillivray T., Pattie A., Deary I.J., Dhillon B. (2005). Retinal vascular image analysis as a potential screening tool for cerebrovascular disease: A rationale based on homology between cerebral and retinal microvasculatures. J. Anat..

[12] Shariflou S., Georgevsky D., Mansour H., Rezaeian M., Hosseini N., Gani F., Gupta V., Braidy N., Golzan S.M. (2017). Diagnostic and prognostic potential of retinal biomarkers in early on-set Alzheimer’s Disease. Curr. Alzheimer Res..

[13] Berisha F., Feke G.T., Trempe C.L., McMeel J.W., Schepens C.L. (2007). Retinal abnormalities in early Alzheimer’s Disease. Investig. Ophthalmol. Vis. Sci..

[14] Frost S., Kanagasingam Y., Sohrabi H., Vignarajan J., Bourgeat P., Salvado O., Villemagne V., Rowe C.C., Macaulay S.L., Szoeke C. (2013). Retinal vascular biomarkers for early detection and monitoring of Alzheimer’s disease. Transl. Psychiatry.

[15] Williams M.A., McGowan A.J., Cardwell C.R., Cheung C.Y., Craig D., Passmore P., Silvestri G., Maxwell A.P., McKay G.J. (2015). Retinal microvascular network attenuation in Alzheimer’s disease. Alzheimer Dement. Diagn. Assess. Dis. Monit..

[16] Ramírez J.M., Rojas B., Gallego B.I., García-Martín E.S., Triviño A., Ramírez A.I., Salazar J.J., de Hoz R. (2014). Glia and blood retinal barrier: Effects of ocular hypertension. Cardiovascular Disease II.

[17] Cogan D.G., Kuwabara T. (1967). The Mural Cell in Perspective. Arch. Ophthalmol..

[18] Bron A.J., Tripathi R.C., Tripathi B.J. (1997). The choroid and uveal vessels. Wolff ’s Anatomy of the Eye and the Orbit.

[19] Bill A. (1975). Blood circulation and fluid dynamics in the eye. Physiol. Rev..

[20] Chan V.T.T., Sun Z., Tang S., Chen L.J., Wong A., Tham C.C., Wong T.Y., Chen C., Ikram M.K., Whitson H.E. (2019). Spectral-Domain OCT measurements in Alzheimer’s Disease: A systematic review and meta-analysis. Ophthalmology.

[21] DeBuc D.C., Gaca M.-W., Grzybowski A., Kanclerz P. (2019). Identification of retinal biomarkers in Alzheimer’s Disease using Optical Coherence Tomography: Recent insights, challenges, and opportunities. J. Clin. Med..

[22] Jáñez L.-E., Jáñez L.-G., Salobrar E.-G., Santos A.-M., de Hoz R., Yubero R., Gil P., Ramírez J.M. (2019). Spatial analysis of thickness changes in ten retinal layers of Alzheimer’s disease patients based on optical coherence tomography. Sci. Rep..

[23] Mihailovic N., Brand C., Lahme L., Schubert F., Bormann E., Eter N., Alnawaiseh M. (2018). Repeatability, reproducibility and agreement of foveal avascular zone measurements using three different optical coherence tomography angiography devices. PLoS ONE.

[24] de la Rosa M.G., Gonzalez-Hernandez M., Sigut J., Alayon S., Radcliffe N., Mendez-Hernandez C., García-Feijoo J., Fuertes-Lazaro I., Perez-Olivan S., Ferreras A. (2013). Measuring hemoglobin levels in the optic nerve head: Comparisons with other structural and functional parameters of glaucoma. Investig. Ophthalmol. Vis. Sci..

[25] Mendez-Hernandez C., Garcia-Feijoo J., Arribas-Pardo P., Saenz-Frances F., Rodriguez-Uña I., Fernandez-Perez C., de la Rosa M.G. (2016). Reproducibility of optic nerve head hemoglobin measures. J. Glaucoma.

[26] Mendez-Hernandez C., Rodriguez-Uña I., Gonzalez-de-la Rosa M., Arribas-Pardo P., Garcia-Feijoo J. (2016). Glaucoma diagnostic capacity of optic nerve head haemoglobin measures compared with spectral domain OCT and HRT III confocal tomography. Acta Ophthalmol..

[27] Nickla D.L., Wallman J. (2010). The multifunctional choroid. Prog. Retin. Eye Res..

[28] Bayhan H.A., Aslan Bayhan S., Celikbilek A., Tanık N., Gürdal C., Tanik N., Gürdal C. (2014). Evaluation of the chorioretinal thickness changes in Alzheimer’s disease using spectral-domain optical coherence tomography. Clin. Exp. Ophthalmol..

[29] Gharbiya M., Trebbastoni A., Parisi F., Manganiello S., Cruciani F., D’Antonio F., De Vico U., Imbriano L., Campanelli A., De Lena C. (2014). Choroidal thinning as a new finding in Alzheimer’s Disease: Evidence from enhanced depth imaging spectral domain Optical Coherence Tomography. J. Alzheimer Dis..

[30] Cunha J.P., Proença R., Dias-Santos A., Melancia D., Almeida R., Águas H., Santos B.O., Alves M., Ferreira J., Papoila A.L. (2017). Choroidal thinning: Alzheimer’s disease and aging. Alzheimer Dement. Diagn. Assess. Dis. Monit..

[31] Trebbastoni A., Marcelli M., Mallone F., D’Antonio F., Imbriano L., Campanelli A., de Lena C., Gharbiya M. (2016). Attenuation of choroidal thickness in patients with Alzheimer Disease: Evidence from an Italian Prospective Study. Alzheimer Dis. Assoc. Disord..

[32] Grewal D.S., Polascik B.W., Hoffmeyer G.C., Fekrat S. (2018). Assessment of differences in retinal microvasculature using OCT angiography in Alzheimer’s disease: A twin discordance report. Ophthalmic Surg. Lasers Imaging Retin..

[33] Bulut M., Yaman A., Erol M.K., Kurtulus F., Toslak D., Dogan B., Turgut Coban D., Kaya Basar E. (2016). Choroidal thickness in patients with mild cognitive impairment and Alzheimer’s type dementia. J. Ophthalmol..

[34] Querques G., Borrelli E., Sacconi R., De Vitis L., Leocani L., Santangelo R., Magnani G., Comi G., Bandello F. (2019). Functional and morphological changes of the retinal vessels in Alzheimer’s disease and mild cognitive impairment. Sci. Rep..

[35] Haan J.D., van de Kreeke J.A., van Berckel B.N., Barkhof F., Teunissen C.E., Scheltens P., Verbraak F.D., Bouwman F.H. (2019). Is retinal vasculature a biomarker in amyloid proven Alzheimer’s disease?. Alzheimer Dement..

[36] Tsai Y., Lu B., Ljubimov A.V., Girman S., Ross-Cisneros F.N., Sadun A.A., Svendsen C.N., Cohen R.M., Wang S. (2014). Ocular changes in TgF344-AD rat model of Alzheimer’s Disease. Investig. Ophthalmol. Vis. Sci..

[37] Kam J.H., Lenassi E., Jeffery G. (2010). Viewing ageing eyes: Diverse sites of amyloid beta accumulation in the ageing mouse retina and the up-regulation of macrophages. PLoS ONE.

[38] Ning A., Cui J., To E., Ashe K., Matsubara J. (2008). Amyloid-β deposits lead to retinal degeneration in a mouse model of Alzheimer Disease. Investig. Ophthalmol. Vis. Sci..

[39] Bailey T.L., Rivara C.B., Rocher A.B., Hof P.R. (2004). The nature and effects of cortical microvascular pathology in aging and Alzheimer’s disease. Neurol. Res..

[40] Marchesi V.T. (2011). Alzheimer’s dementia begins as a disease of small blood vessels, damaged by oxidative-induced inflammation and dysregulated amyloid metabolism: Implications for early detection and therapy. FASEB J..

[41] Miao J., Xu F., Davis J., Otte-Höller I., Verbeek M.M., Van Nostrand W.E. (2005). Cerebral microvascular amyloid β protein deposition induces vascular degeneration and neuroinflammation in transgenic mice expressing human vasculotropic mutant amyloid β precursor protein. Am. J. Pathol..

[42] Cheung C.Y.L., Ong Y.-T.T., Ikram M.K., Chen C., Wong T.Y. (2014). Retinal microvasculature in Alzheimer’s disease. J. Alzheimer Dis..

[43] Golzan S.M., Goozee K., Georgevsky D., Avolio A., Chatterjee P., Shen K., Gupta V., Chung R., Savage G., Orr C.F. (2017). Retinal vascular and structural changes are associated with amyloid burden in the elderly: Ophthalmic biomarkers of preclinical Alzheimer’s disease. Alzheimer Res. Ther..

[44] Jiang H., Wei Y., Shi Y., Wright C.B., Sun X., Gregori G., Zheng F., Vanner E.A., Lam B.L., Rundek T. (2018). Altered macular microvasculature in mild cognitive impairment and Alzheimer Disease. J. Neuro-Ophthalmol..

[45] Lahme L., Esser E.L., Mihailovic N., Schubert F., Lauermann J., Johnen A., Eter N., Duning T., Alnawaiseh M. (2018). Evaluation of ocular perfusion in Alzheimer’s Disease using Optical Coherence Tomography Angiography. J. Alzheimer Dis..

[46] O’bryhim B., Apte R., Kung N., Coble D., Van Starven G.P. (2018). Association of preclinical Alzheimer disease with optical coherence tomographic angiography findings. JAMA Ophthalmol..

[47] Van De Kreeke J.A., Nguyen H.T., Konijnenberg E., Tomassen J., Den Braber A., Ten Kate M., Yaqub M., Van Berckel B., Lammertsma A.A., Boomsma D.I. (2019). Optical coherence tomography angiography in preclinical Alzheimer’s disease. Br. J. Ophthalmol..

[48] Cioffi G., Granstam E., Kaufman P.L., Alm A. (2004). Circulación ocular. Adler Fisiología Del Ojo.

[49] Ramírez J.M., Ramírez A.I., Salazar J.J., de Hoz R., Rojas B., Triviño A., Mones J., Gómez-Ulla F. (2005). Anatomofisiología de la úvea posterior: Coroides. Degeneracion Macular Asociada A La Edad.

[50] Delaey C., Van de Voorde J. (2000). Regulatory mechanisms in the retinal and choroidal circulation. Ophthalmic Res..

[51] Yu D.-Y., Alder V.A., Cringle S.J., Brown M.J. (1988). Choroidal blood flow measured in the dog eye in vivo and in vitro by local hydrogen clearance polarography: Validation of a technique and response to raised. Exp. Eye Res..

[52] Alm A., Bill A. (1973). Ocular and optic nerve blood flow at normal and increased intraocular pressures in monkeys (Macaca irus): A study with radioactively labelled microspheres including. Exp. Eye Res..

[53] Alm A., Bill A. (1970). Blood flow and oxygen extraction in the cat uvea at normal and high intraocular pressures. Acta Physiol. Scand..

[54] Bill A. (1974). Effects of acetazolamide and carotid occlusion on the ocular blood flow in unanesthetized rabbits. Investig. Ophthalmol. Vis. Sci..

[55] Friedman E. (1970). Choroidal blood flow: Pressure-flow relationships. Arch. Ophthalmol..

[56] Geijer C., Bill A. (1979). Effects of raised intraocular pressure on retinal, prelaminar, laminar, and retrolaminar optic nerve blood flow in monkeys. Investig. Ophthalmol. Vis. Sci..

[57] Takats I., Leiszter F. (1979). Relationship between blood flow velocity in the choroid and intraocular pressure in rabbits. Acta Ophthalmol..

[58] Triviño A., de Hoz R., Salazar J.J., Ramírez A.I., Rojas B., Ramírez J.M. (2002). Distribution and organization of the nerve fiber and ganglion cells of the human choroid. Anat. Embryol. Berl..

[59] Triviño A., de Hoz R., Rojas B., Salazar J.J., Ramirez A.I., Ramirez J.M. (2005). NPY and TH innervation in human choroidal whole-mounts. Histol. Histopathol..

[60] Schmidt R.E., Beaudet L.N., Plurad S.B., Dorsey D.A. (1997). Axonal cytoskeletal pathology in aged and diabetic human sympathetic autonomic ganglia. Brain Res..

[61] Kaarniranta K., Salminen A., Haapasalo A., Soininen H., Hiltunen M. (2011). Age-related macular degeneration (AMD): Alzheimer’s disease in the eye?. J. Alzheimer Dis..

[62] Diomedi M., Misaggi G. (2013). Vascular contribution to Alzheimer disease: Predictors of rapid progression. CNS Neurol. Disord. Drug Targets.

[63] Wong T.Y., Klein R., Klein B.E.K., Tielsch J.M., Hubbard L., Nieto F.J. (2001). Retinal microvascular abnormalities and their relationship with hypertension, cardiovascular disease, and mortality. Surv. Ophthalmol..

[64] Crittin M., Riva C. (2004). Functional imaging of the human papilla and peripapillary region based on flicker-induced reflectance changes. Neurosci. Lett..

[65] Pena-Betancor C., Gonzalez-Hernandez M., Fumero-Batista F., Sigut J., Medina-Mesa E., Alayon S., Gonzalez de la Rosa M. (2015). Estimation of the relative amount of hemoglobin in the cup and neuroretinal rim using stereoscopic color fundus images. Investig. Ophthalmol. Vis. Sci..

[66] Medina-Mesa E., Gonzalez-Hernandez M., Sigut J., Fumero-Batista F., Pena-Betancor C., Alayon S., Gonzalez de la Rosa M. (2016). Estimating the amount of hemoglobin in the neuroretinal rim usingcColor images and OCT. Curr. Eye Res..

[67] Gonzalez-Hernandez M., Sigut Saavedra J., Gonzalez de la Rosa M. (2017). Relationship between retinal nerve fiber layer thickness and hemoglobin present in the optic nerve head in glaucoma. J. Ophthalmol..

[68] Bambo M.P., Garcia-Martin E., Perez-Olivan S., Sigut J., Fumero F., Fuentes J.L., Ara J.R., Martin J., Larrosa J.M., Gonzalez de la Rosa M. (2013). Diagnostic ability of a new method for measuring haemoglobin levels in the optic nerve head in multiple sclerosis patients. Br. J. Ophthalmol..

[69] Bambo M.P., Garcia-Martin E., Satue M., Perez-Olivan S., Alayon S., Gonzalez-Hernandez M., Polo V., Larrosa J.M., Gonzalez de la Rosa M. (2014). Measuring hemoglobin levels in the optic disc of Parkinson’s disease patients using new colorimetric analysis software. Parkinsons. Dis..

[70] Bambo M.P., Garcia-Martin E., Gutierrez-Ruiz F., Pinilla J., Perez-Olivan S., Larrosa J.M., Polo V., Pablo L. (2015). Analysis of optic disk color changes in Alzheimer’s disease: A potential new biomarker. Clin. Neurol. Neurosurg..

